# Exploring Nursing Team Perceptions of Sleep in Critically Ill Pediatric Patients: A Mixed-Methods Study

**DOI:** 10.3390/children13081035

**Published:** 2026-08-03

**Authors:** Alicia Gomez-Merino, Paloma M. Núñez-Yebra, Rafael Lobato-López, Natalia González-Martínez, Elena García-González, Pedro Piqueras-Rodríguez, Desiree Alcaraz-Blanco, Marta Romeral-Jiménez, Marta Martín-Velasco, Patricia Luna-Castaño

**Affiliations:** 1 Pediatric Intensive Care Unit, La Paz University Hospital, 28046 Madrid, Spain; agomez2719@alumno.uned.es (A.G.-M.); mpaloma.nunez@salud.madrid.org (P.M.N.-Y.); rafael.lobato@salud.madrid.org (R.L.-L.); ngonzalezmartinez@salud.madrid.org (N.G.-M.); elena.garcia.gonzalez@uvic.cat (E.G.-G.); pedro.piqueras@salud.madrid.org (P.P.-R.); desire.alcaraz@salud.madrid.org (D.A.-B.); marta.romeral@salud.madrid.org (M.R.-J.); mmvelasco@salud.madrid.org (M.M.-V.); 2 Hospital La Paz Institute for Health Research—IdiPAZ (La Paz University Hospital—Universidad Autónoma de Madrid—Getafe Universitary Hospital—Universidad Europea de Madrid), 28046 Madrid, Spain; 3 Doctoral Program in Biomedical Sciences and Public Health, National Distance Education University (UNED), 28015 Madrid, Spain; 4 Department of Nursing, Villanueva de la Cañada Campus, Alfonso X el Sabio University, 28691 Madrid, Spain; 5 Department of Nursing, Autonomous University of Madrid, 28049 Madrid, Spain; 6 PhD Programme in Comprehensive Care and Health Services, Doctoral School, University of Vic-Central University of Catalonia (UVic-UCC), 08500 Vic, Spain; 7 Health Care Research Group, Hospital La Paz Institute for Health Research—IdiPAZ (La Paz University Hospital—Universidad Autónoma de Madrid—Getafe Universitary Hospital—Universidad Europea de Madrid), 28046 Madrid, Spain; 8 Health Care and Services Research Unit (Investén-ISCIII), 28029 Madrid, Spain

**Keywords:** nursing, pediatric intensive care units, pediatrics, sleep disorders, sleep quality

## Abstract

**Highlights:**

**What are the main findings?**

- Across both qualitative and quantitative findings, noise, light, nighttime interventions, and delirium were consistently perceived as key sleep-disrupting factors in the PICU.
- The nursing staff has a widespread perception that sleep is poor in the PICU, making greater awareness among professionals essential.

**What are the implications of the main findings?**

- Identifying factors that disrupt sleep in the PICU allows for the development of specific strategies for managing them.
- Raising awareness among nursing staff about sleep in critically ill pediatric patients will strengthen the implementation of measures that promote rest.

**Abstract:**

Objective: To explore nursing team perceptions of sleep and sleep-disrupting factors in critically ill pediatric patients. Methodology: Convergent mixed-methods study comprising an exploratory qualitative and descriptive cross-sectional quantitative. For the qualitative component, the population consisted of the PICU nursing team with ≥3 years of experience until theoretical saturation was reached. Semi-structured interviews were conducted regarding their perceptions of sleep in critically ill children. Data were analyzed using transcription, immersive reading, and coding. For the quantitative component, the population was selected using convenience sampling without exclusion. The variables were years of experience, professional category, and factors affecting rest. Data were collected using an ad hoc Likert-type questionnaire. For the analysis, the median and interquartile range were calculated for quantitative variables, and frequencies and percentages were calculated for categorical variables. Bivariate analysis was performed using the Mann-Whitney U test. Results: Ten interviews were conducted, revealing three main categories: “Factors Affecting Sleep,” with noise, nighttime interventions, and light as the most frequent codes; “Consequences of Sleep Disturbances,” with delirium and recovery as the most frequent codes; and “Perception of Sleep in the PICU and Need for Professional Awareness”. For the quantitative results, 78.3% (n = 65) of the nursing team participated. Among the factors affecting rest, the most relevant were “Inadequately controlled pain,” identified as “Highly relevant” by 84.6%, and “Delirium,” “Withdrawal syndrome,” “Light,” “Nocturnal environmental noise,” and “Nighttime interventions/procedures,” identified as “Highly relevant” by over 70%. In contrast, “Continuous nocturnal glucose administration” was identified as “Slightly/Not relevant” by almost 40%. Differences between nurses and nursing assistants were observed regarding the perceived relevance of nocturnal environmental noise, delirium, physical restraints and continuous nocturnal glucose administration (*p* ≤ 0.05). Conclusions: Qualitative and quantitative results concur in identifying, from the perspective of nursing professionals in a single PICU, noise, light, interventions, and delirium as negative factors for rest.

## 1. Introduction

Sleep is a fundamental biological process and a central pillar for healthy development in childhood, acting as a regulator in the maturation of the central nervous system and physiological balance [1]. Scientific evidence has demonstrated that adequate sleep, both in quantity and quality, is necessary for proper cognitive, emotional, physical, and psychomotor development [2].

From a neurocognitive perspective, during the distinct stages of sleep, especially during REM sleep, the brain processes, organizes, and stabilizes information acquired during wakefulness, promoting learning, attention, concentration, and long-term memory [1,2,3].

In addition to its cognitive relevance, sleep plays a fundamental role in emotional and behavioral regulation, promoting mood stability and social adaptation [2,3]. Similarly, sleep disorders have been identified as a risk factor for the emergence of behavioral problems, including behaviors such as aggression, hyperactivity, or difficulties in self-regulation [1,2].

Furthermore, sleep is also a key element for growth and physical health [2,4]. Particularly, during non-REM sleep, important processes of tissue repair, organ maturation, and immune regulation occur, accompanied by the release of growth hormone [1]. Furthermore, available evidence shows a close relationship between insufficient sleep duration and an increased risk of metabolic disorders, including childhood obesity, insulin resistance, and hormonal imbalances [4].

Finally, sleep contributes significantly to psychomotor development. During rest, motor skills acquired during the day are consolidated, promoting motor coordination, sensory integration, and the refinement of complex movements [1].

Despite the importance of sleep for the pediatric population, hospitalized children, who are especially vulnerable, experience significant disruptions in their sleep patterns, particularly those in Pediatric Intensive Care Units (PICUs) [5].

Several studies have shown that critically ill children exhibit marked disruption of sleep architecture. Among the most relevant characteristics are sleep fragmentation, with multiple awakenings and micro-arousals, which hinder the continuity of rest and the completion of physiological sleep cycles [5,6]. In fact, a significant reduction and even disappearance of deep sleep and REM sleep stages have been described [7]. Furthermore, sleep organization is also affected, resulting in a more homogeneous distribution of sleep-wake cycles throughout the day instead of being predominant during nighttime hours [5].

Regarding the causes of sleep disturbances in critically ill children, their origin is multifactorial, with the PICU environment being a significant factor. This environment is characterized by continuous noise, excessive exposure to artificial light at night, and numerous care interventions performed during the night, such as vital sign monitoring, medication administration, and diagnostic procedures [5,7].

Furthermore, certain common treatments in PICUs, such as sedation or mechanical ventilation, contribute to both sleep fragmentation and disruption of its physiological architecture [5,7]. Finally, intrinsic factors related to the critical illness itself, such as pain, fear, anxiety, stress, and the severity of the clinical process, represent additional barriers to achieving adequate rest [8].

The consequences of sleep disturbances significantly affect the clinical course of critically ill children. Available evidence indicates a close relationship between sleep disruption and the development of delirium, establishing a bidirectional relationship in which both conditions reinforce each other. Of note is the increased morbidity and mortality among patients who experience delirium during their stay in the PICU [6,7]. Furthermore, sleep deprivation is associated with disruption of the hypothalamic-pituitary-adrenal axis, manifested by immunological alterations, an increased inflammatory response, and metabolic disturbances, negatively impacting recovery [7]. Beyond the hospitalization period, sleep disturbances can persist after discharge and contribute to the so-called Pediatric Intensive Care Syndrome (PICS-p), characterized by the development of medium- and long-term cognitive, emotional, and physical sequelae, which have a very negative impact on the quality of life of the child and their family. In fact, several studies have described a high prevalence of sleep disturbances during the weeks following discharge from the critical care unit [5,7,9].

Therefore, given the high prevalence of sleep disturbances in PICU patients and their associated potential consequences, early identification and the implementation of specific strategies aimed at preserving rest have become healthcare priorities. In this context, nursing staff play a fundamental role due to their presence with the patient and their family 24 h a day.

Nurses are uniquely positioned to identify factors that interfere with rest and develop interventions aimed at minimizing their impact [6,10,11]. This includes controlling noise levels, optimizing lighting according to circadian rhythms, and creating a physically comfortable environment that facilitates the child’s sleep, among other measures [6,10,12,13].

Furthermore, organizing care is one of the most effective strategies for reducing sleep fragmentation in PICUs [10,11]. Grouping clinical procedures during specific periods of the day reduces the number of nighttime interruptions, promoting longer rest periods [11]. In fact, several studies indicate that a sizable proportion of awakenings in critically ill patients are related to care activities [11,12].

Along with environmental and organizational modifications, nursing staff can lead the implementation of non-pharmacological interventions aimed at promoting sleep, including relaxation techniques, therapeutic massage, and music therapy [14]. Additionally, parental involvement in bedtime routines provides emotional security for the child and promotes continuity of family routines at home [9,12].

Although healthcare professionals generally recognize that sleep is an essential component of physical and emotional recovery, several studies have described limitations in their knowledge of sleep physiology, its clinical implications, and the most appropriate strategies for its preservation in intensive care settings [15,16,17].

Finally, the use of validated sleep assessment tools, along with the systematic recording of environmental and clinical factors associated with sleep disruptions, facilitates the development of individualized care plans aimed at optimizing patient rest [10,11,12].

Overall, the available evidence suggests that nursing-led interventions, especially when implemented through structured programs or care bundles, can significantly contribute to improving the sleep quality of hospitalized children [11]. However, the effectiveness of these strategies depends largely on the attitudes and perceptions of the professionals responsible for their implementation.

The perceptions and beliefs of healthcare professionals also influence the importance they place on rest within the care process. In intensive care units, where complex clinical situations predominate, sleep may be relegated to a secondary position compared to other priorities considered more urgent. Furthermore, care routines often take precedence over individual rest needs, so care planning is usually organized around the needs of healthcare professionals [12,15,17]. In addition, the systematic assessment of sleep quality remains poorly integrated into practice, meaning that sleep disorders are often underdiagnosed [10,15].

Furthermore, although research on sleep in critically ill patients has grown considerably in recent years, most of the evidence has focused on the adult population, with few studies in the PICU setting. This knowledge gap makes it difficult to understand how professionals interpret this problem, making it necessary to delve deeper into the perspective of nursing staff, given their continuous proximity to the patient and their fundamental role in identifying potentially modifiable factors that impact children’s sleep.

Therefore, this study aimed to explore nursing team perceptions of sleep in critically ill pediatric patients, including factors affecting sleep, perceived consequences of sleep disturbances, and the need for greater professional awareness.

## 2. Materials and Methods

### 2.1. Study Design

A convergent mixed-methods study was conducted, integrating an exploratory qualitative methodology with a cross-sectional descriptive quantitative approach.

The qualitative phase focused on exploring the experiences, beliefs, and meanings attributed by healthcare professionals to sleep in critically ill children, while the quantitative phase complemented these findings through the structured collection of information on the perceived relevance of distinct factors related to rest.

Both study components were developed and conducted in parallel to address the same research objective. Qualitative and quantitative data were analyzed independently using the corresponding analytical approaches. The findings were subsequently integrated during the interpretation stage through a systematic comparison of both datasets to identify areas of convergence and divergence, enabling a more comprehensive understanding of nursing professionals’ perceptions of sleep in critically ill pediatric patients. In addition, to ensure comprehensive and transparent reporting, the qualitative component was reviewed against the Consolidated Criteria for Reporting Qualitative Research (COREQ) checklist prior to resubmission.

### 2.2. Setting and Study Population

The study was conducted in the Pediatric Intensive Care Unit of La Paz University Hospital, Spain, between February and May 2025. The study population consisted of the unit’s nursing team, including nurses and nursing assistants (Spanish acronym: TMSCAE).

For the qualitative phase, purposive sampling was used, selecting nursing professionals with at least three years of experience in the PICU. This criterion was established to ensure that participants had sufficient experience in the care of critically ill pediatric patients and could provide a comprehensive perspective on the factors affecting their rest. The sample size was determined using the theoretical data saturation criterion.

For the quantitative phase, convenience sampling was applied, inviting the entire nursing team of the unit to participate. No exclusion criteria based on years of experience were established to capture the overall perception of all professionals.

### 2.3. Data Collection Instruments

For the qualitative component, a semi-structured interview was designed, the script of which was developed based on a review of the literature on sleep in critically ill patients and the research team’s expertise in pediatric intensive care.

The interviews included open-ended questions aimed at exploring the participants’ professional experience, the importance attributed to sleep in the recovery of critically ill children, the perceived consequences of sleep disturbances, the factors that negatively affect rest in the PICU, the nursing team’s level of awareness regarding this issue, the existing barriers to promoting sleep, the measures routinely implemented to promote rest, and potential improvement strategies. A final open-ended question was also included to identify aspects that the researchers had not considered.

For the quantitative component, an ad hoc questionnaire was designed based on a prior literature review conducted by the research team. The review included studies examining factors affecting rest in critically ill patients, as well as previous research addressing healthcare professionals’ perceptions of sleep-related factors in critical care settings [5,7,15,17]. Once the initial version had been developed, the questionnaire was pilot-tested with eight members of the nursing team: four nurses and four nursing assistants (TMSCAE). The pilot testing aimed to assess the clarity, comprehensibility, and apparent comprehensiveness of the questionnaire items and response options. Following this process, the research team determined that no modifications were required. The questionnaires completed during the pilot testing were excluded from the final analysis.

This questionnaire consisted of 16 items, divided into two sections. The first section included sociodemographic variables (professional category and years of experience in the PICU), and the second section assessed factors potentially involved in the development of sleep disturbances, evaluating each item using a four-point Likert scale, from “Highly relevant” to “Not relevant,” with an additional “Do not know/No response” option. The factors collected included: environmental factors, clinical and pharmacological factors, care and organizational factors, and factors related to the patient’s clinical complexity.

### 2.4. Data Collection

The qualitative phase was conducted through face-to-face, individual interviews carried out by members of the research team who had been previously trained in qualitative interviewing techniques. Each interview lasted approximately 15–20 min. Audio recordings were also made with the participants’ prior consent.

For the quantitative component, the questionnaire was distributed through an anonymous and voluntary electronic survey during the same study period. Both study components were conducted independently and in parallel.

### 2.5. Data Analysis

The qualitative data were analyzed using an inductive thematic approach. The analytical process was consistent with the stages described by Braun and Clarke and the trustworthiness criteria proposed by Nowell et al. [18,19]. First, the interviews were transcribed verbatim, and both researchers became familiar with the data through repeated and immersive reading of the transcripts. The ten interviews were then independently coded in full by two researchers using QDA Miner Lite (Provalis Research, Montreal, QC, Canada). Initial codes were generated inductively and subsequently grouped into conceptually related subcategories and three overarching categories through an iterative process of constant comparison.

Following the independent coding process, the researchers compared the codes assigned to the transcripts. Differences in coding or interpretation were discussed by revisiting the relevant excerpts and their broader narrative context until consensus was reached. When necessary, code definitions were clarified, merged, or refined. The resulting subcategories and main categories were subsequently reviewed against the coded extracts and the complete dataset, defined, named, and supported by representative verbatim quotations. Code frequencies were defined as the number of coded excerpts across all ten interview transcripts. Since a single participant could contribute more than one excerpt to the same code, these frequencies should not be interpreted as the number or proportion of participants who mentioned each topic.

Regarding the analysis of quantitative data, the median and interquartile range (IQR) were calculated for quantitative variables, given the non-normal nature of their distribution. Categorical variables were expressed using absolute frequencies and percentages. Each questionnaire item was analyzed independently. Responses to the Likert-scale items were treated as ordinal variables (“Not relevant” = 1 to “Highly relevant” = 4). “Do not know/No response” responses were considered missing values for the corresponding item and were excluded only from that specific analysis. Regarding the inferential analysis, differences in the perceived relevance of the different sleep-related factors according to professional category were analyzed using the Mann–Whitney U test. A *p*-value ≤ 0.05 was considered statistically significant.

### 2.6. Ethical Considerations

Participation in the study was voluntary. In the qualitative phase, interviews were recorded after obtaining the participants’ consent. The information collected was treated confidentially, ensuring the anonymization of the discourse during the transcription, analysis, and presentation of results. The data were used exclusively for research purposes.

## 3. Results

### 3.1. Qualitative Results

A total of 10 interviews were conducted, at which point theoretical data saturation was reached. Seven nurses and three nursing assistants participated, with professional experience in the PICU ranging from 3 to 22 years.

Thematic analysis revealed three main categories: (1) Factors affecting sleep, (2) Consequences of sleep disorders, and (3) Perception of sleep in the PICU and need for awareness.

#### 3.1.1. Category 1: Factors Affecting Sleep

Participants identified multiple factors capable of disrupting the sleep of critically ill children. Based on the number of coded excerpts, the most frequently occurring codes were: noise (32 excerpts), work routines (29 excerpts), nursing interventions (20 excerpts), and light (15 excerpts). Other factors mentioned included parental presence, circadian rhythm disturbances, sedation, pain, and certain pharmacological treatments.

Furthermore, ambient noise was identified as one of the main sleep disruptors, especially noise from alarms, infusion pumps, and nighttime care activities: “The noises. They complain about the light, but I would say the beeping, which also means you are constantly going in and out of the room.”

Care interventions and certain organizational routines were also perceived as a significant source of sleep disruptions: “Every time we go in at night, whether it is necessary or not, we interrupt sleep. I believe we are not always aware of the importance of grouping care.

Several participants also indicated that certain routine activities could be reconsidered: “Sometimes we insist on doing things that disrupt children’s sleep.”

#### 3.1.2. Category 2: Consequences of Sleep Disturbances

Professionals consistently associated sleep deprivation with worse clinical outcomes, delayed recovery, and the development of delirium. The most frequently occurring codes were: delirium (9 excerpts), recovery (8 excerpts), and complications (7 excerpts).

Furthermore, participants considered sleep to play a significant role in both the child’s physical and emotional recovery: “Very important. If they suffer from sleep disturbances, I think it affects the prognosis and recovery.

In addition, delirium was repeatedly identified as one of the main consequences of sleep disturbances: “I think this greatly affects delirium. Not sleeping at night makes them disoriented, and that affects the child’s improvement.”

#### 3.1.3. Category 3: Perception of Sleep in the PICU and Need for Awareness

This category comprised two interrelated subcategories: the nursing team’s overall perception of sleep quality in the PICU and the perceived need for greater professional awareness and the implementation of improvement strategies.

Subcategory 3.1. Overall Perception of Sleep in the PICU: Across both professional groups, participants expressed a general perception that pediatric patients do not achieve adequate rest during their PICU stay: “So-so. They sleep better now than before, but we still cause many unnecessary awakenings.”

Subcategory 3.2. Need for Professional Awareness and Improvement Strategies: The need for greater professional awareness emerged as one of the most recurrent findings during the interviews, with awareness representing the most frequently occurring code within this category. Although participants acknowledged improvements in sleep protection, they also identified opportunities for improvement at both the individual and organizational levels: “We are not aware of the issue, either individually or as a team. We need to change our routines and our mindset”; “We need to talk more about sleep and make people aware of its importance.”

Participants also proposed several measures aimed at improving rest, including clustering care activities, reducing noise, increasing the flexibility of certain organizational routines, adapting treatment schedules, and promoting greater family involvement in care.

### 3.2. Quantitative Results

Of the 83 nursing team members invited to participate, 65 completed the questionnaire, corresponding to a response rate of 78.3%. Of these, 66.2% (n = 43) were completed by nurses and 33.8% (n = 22) by nursing assistants. The median professional experience in the PICU was 5 years (IQR = 7).

Regarding the factors most frequently identified as “highly relevant” to the development of sleep disturbances, these were withdrawal syndrome (87.7%), inadequately controlled pain (84.6%), delirium (78.5%), nighttime interventions/procedures performed by healthcare professionals (72.3%), nocturnal environmental noise (70.8%), limited exposure to natural light during the day (69.2%), and exposure to artificial light at night (64.6%).

Conversely, factors such as continuous glucose administration overnight or corticosteroid administration outside the physiological cortisol secretion period were perceived as less relevant. Table 1 details the participants’ assessments of each of the factors collected.

However, in the exploratory analysis, significant differences were identified between professional categories and the assessment of some factors. Nurses and nursing assistants differed in the importance attributed to nocturnal environmental noise (*p* = 0.001), physical restraints (*p* = 0.011), delirium (*p* < 0.001), and continuous glucose administration at night (*p* = 0.001) (Table 2).

Regarding the integration of qualitative and quantitative results, a high degree of convergence between both methodological approaches was demonstrated. This was reflected in the fact that, in both phases, noise, light, nighttime interventions, and delirium were identified as determining factors affecting the sleep of critically ill pediatric patients. Furthermore, the professionals linked sleep disturbances to poorer clinical recovery and emphasized the need to raise awareness among the healthcare team regarding the importance of rest. Following the independent analyses of the qualitative and quantitative data, the findings from both components were systematically compared during the interpretation stage to identify areas of convergence and divergence. Integrating both approaches allowed for a more comprehensive understanding of the phenomenon, contributing both the professionals’ perceptions and experiences, as well as the perceived relevance of the different sleep-related factors (Table 3).

## 4. Discussion

Critically ill patients frequently experience sleep disturbances during their hospital stay [5,20,21,22], a finding that aligns closely with the perception of rest held by the participants in this study: they sleep… “poorly, better than before, but poorly.”

In this regard, consistent with the results reported by Alfadhel et al. [23], although the nursing team considers sleep a key aspect of the recovery of critically ill pediatric patients, there are still multiple factors stemming from the healthcare environment and/or the care itself that pose a significant barrier to ensuring adequate nighttime rest. Similarly, the study by Tas Arslan et al. [17] reached comparable conclusions, although that study focused on nursing professionals caring for neonatal patients.

Regarding the environmental factors specific to an ICU, the results of this study show that noise, followed by light, has the greatest impact on nighttime rest. These variables have been extensively studied, and in line with the present results, there is a direct correlation between noise in an ICU and sleep deprivation [7,22,24]. Regarding light, this study reveals that, for the nursing staff, avoiding light pollution at night is just as important as adequate exposure to natural light during the day, which also coincides with the available literature that recommends appropriate light-dark alternation to achieve alignment of circadian rhythms [7,8,22].

Another factor identified as disrupting sleep by the participants in this study is nighttime nursing interventions and work routines. In this respect, studies such as those by Locihova et al. [25] and Gomez-Merino et al. [26] indicate that many of the nighttime interventions performed are unnecessary or are conducted due to routines inherent in the hospital environment rather than the actual needs of the patients.

Regarding the clinical aspects revealed in this study which can interfere with rest, withdrawal syndrome, pain, and/or delirium stood out.

In this respect, the routine use of analgesic-sedative medications such as opioids or benzodiazepines in the PICU, and the associated withdrawal syndrome, were perceived by the nursing team as a major disruptive factors to rest, an aspect widely supported by the reviewed literature [5,7,14].

Regarding pain, numerous studies reveal that it acts as a powerful factor contributing to sleep disturbances and that, in turn, sleep deprivation increases a child’s sensitivity to pain, thus becoming one of the main barriers to adequate rest [10,15,16]. These results coincide with the present study, in which virtually 100% of participants considered pain a disruptive factor affecting sleep.

Concerning delirium, the available scientific evidence aligns with these results, describing a strong, direct, and bidirectional relationship between sleep disturbances and delirium. It shows that patients suffering from delirium experience much more fragmented sleep and a severe reduction in REM and slow-wave sleep, crucial sleep phases for cognitive restoration and brain plasticity [6,7,27].

In addition to all these variables that disrupt sleep, there are others, such as nocturnal glucose administration or the administration of corticosteroids at times other than the physiological secretion of cortisol, which are also identified in the literature as significant sleep disruptors [28,29,30]. However, in contrast, these variables were not perceived by the participants in this study as highly relevant.

Nocturnal glucose administration, the use of nocturnal intravenous fluid therapy, or continuous enteral nutrition administered via a gastric tube are common practices in Pediatric Intensive Care Units. Glucose administration is considered a regulator of circadian rhythms and, therefore, uninterrupted administration, including at night, promotes the dysregulation of all internal biorhythms, also affecting the sleep-wake cycle [28,29].

On the other hand, administering corticosteroids outside the physiological circadian pattern promotes the desynchronization of circadian rhythms. In other words, glucocorticoids also function as regulators of biological rhythms and, therefore, administration at the wrong time disrupts circadian cycles [30].

An important finding of this study is the potential gap between the factors documented in the literature to disrupt sleep and the relevance attributed to them by the nursing team. Pain, withdrawal syndrome, delirium, noise, and nighttime interventions were widely recognized as relevant, whereas less immediately observable factors, such as continuous nocturnal glucose administration and corticosteroid administration outside the physiological schedule, received lower relevance ratings. This discrepancy may indicate that environmental and symptomatic factors are more readily recognized than factors involving circadian regulation, treatment timing, or routine clinical practices. The differences observed between nurses and nursing assistants in the perceived relevance of several factors may suggest the value of considering the specific roles and responsibilities of each professional group when designing educational strategies. Nevertheless, as this study assessed perceptions rather than objective knowledge, these findings should be interpreted as a potential perception-to-evidence gap rather than as evidence of insufficient professional competence.

This gap is particularly relevant because many of the identified factors are potentially modifiable through education and changes in daily clinical practice. Educational interventions could strengthen professionals’ awareness of sleep physiology, circadian regulation, and the environmental, clinical, and care-related factors that may disrupt sleep [15,16,17]. In clinical practice, this knowledge could be translated into structured sleep-promotion protocols incorporating non-pharmacological interventions and environmental modifications [10,12,14]. Specific measures may include clustering care activities and reducing unnecessary nighttime interventions [25,26], optimizing noise and light exposure [10,12,14], reviewing the timing of medications and nutritional support when clinically feasible [28,29,30], promoting family involvement in bedtime routines [11,14], and systematically assessing sleep quality. Integrating these measures into interdisciplinary protocols may help establish sleep protection as an explicit component of routine PICU care.

Given that many sleep-disrupting factors are modifiable, identifying differences between evidence-based recommendations and professionals’ perceptions represents an essential first step toward designing targeted and clinically feasible sleep-promotion strategies. Future multicenter studies will be important to determine whether these findings are consistent across different PICU settings.

### Study Limitations

This study has several limitations to be considered when interpreting the results. First, it was conducted in a single Pediatric Intensive Care Unit, which limits the transferability of the results. The perceptions identified may be influenced by the organizational culture, staffing characteristics, and local clinical practices of this institution and, therefore, should not be assumed to represent those of other PICUs.

Although the ad hoc questionnaire was based on a literature review and pilot-tested with the target population, its content validity was not formally assessed. Furthermore, as the questionnaire comprised conceptually distinct factors that were analyzed individually rather than as a unidimensional scale, the calculation of an overall internal consistency coefficient, such as Cronbach’s alpha, was not applicable. In addition, as the quantitative analysis was exploratory, no adjustment for multiple comparisons was applied; therefore, the statistically significant differences observed between professional groups should be interpreted cautiously. These considerations should be taken into account when interpreting the quantitative findings.

Regarding the qualitative phase, although theoretical data saturation was reached, the interpretation of the discourses may be influenced by the subjectivity inherent in qualitative analysis. However, this risk was minimized through independent coding by two researchers and the consensual resolution of discrepancies.

Finally, future lines of research should include multicenter studies that, in addition to evaluating professionals’ perceptions, combine this with objective sleep assessment indicators, which could provide a more comprehensive understanding of the phenomenon.

## 5. Conclusions

The qualitative findings indicate that nursing professionals generally perceive that critically ill pediatric patients do not achieve adequate rest during their PICU stay. Noise, light, nighttime interventions, and delirium emerged as relevant sleep-disrupting factors across both study components. In the quantitative findings, withdrawal syndrome and inadequately controlled pain received the highest ratings of perceived relevance. Furthermore, a lack of awareness and sensitivity regarding the importance of sleep for the recovery of critically ill children was one of the most frequently mentioned concerns among participants.

On the other hand, the discrepancies among the nursing team regarding the factors that affect rest highlight the value of studies as this one, which explore nursing perceptions. These findings may facilitate the implementation of specific strategies that promote respect for nighttime rest and are based on previously identified needs, while providing a basis for future multicenter studies to determine whether these perceptions are consistent across different PICU settings.

## Figures and Tables

**Table 1:** Distribution of responses regarding the relevance of factors associated with sleep disturbances in critically ill pediatric patients.

Domain/Factor	Highly Relevant n (%)	Relevant n (%)	Slightly Relevance n (%)	Not Relevant n (%)	DK/DR n (%)
ENVIRONMENTAL FACTORS					
Artificial light exposure at night	42 (64.6)	16 (24.6)	5 (7.7)	2 (3.1)	0
Limited exposure to natural daylight	45 (69.2)	14 (21.5)	4 (6.2)	2 (3.1)	0
Nocturnal environmental noise	46 (70.8)	19 (29.2)	0	0	0
Limited family involvement	25 (38.5)	34 (52.3)	6 (9.2)	0	0
CLINICAL AND PHARMACOLOGICAL FACTORS	Highly relevant n (%)	Relevant n (%)	Slightly Relevance n (%)	Not Relevant n (%)	DK/DR n (%)
Inadequately controlled pain	55 (84.6)	8 (12.3)	1 (1.5)	1 (1.5)	0
Intravenous sedation	21 (32.3)	35 (53.8)	4 (6.2)	2 (3.1)	3 (4.6)
Corticosteroid administration outside the physiological schedule	5 (7.7)	33 (50.8)	14 (21.5)	2 (3.1)	11 (16.9)
Delirium	51 (78.5)	11 (16.9)	1 (1.5)	2 (3.1)	0
Withdrawal syndrome	57 (87.7)	7 (10.8)	0	0	1 (1.5)
CARE-RELATED AND ORGANIZATIONAL FACTORS	Highly relevant n (%)	Relevant n (%)	Slightly Relevance n (%)	Not Relevant n (%)	DK/DR n (%)
Nighttime interventions/procedures	47 (72.3)	15 (23.1)	3 (4.6)	0	0
Nighttime administration of diuretics/enemas	39 (60.0)	17 (26.2)	5 (7.7)	4 (6.2)	0
FACTORS RELATED TO THE PATIENT’S CLINICAL COMPLEXITY	Highly relevant n (%)	Relevant n (%)	Slightly Relevance n (%)	Not Relevant n (%)	DK/DR n (%)
Physical restraints	17 (26.2)	37 (56.9)	10 (15.4)	0	1 (1.5)
Multiple invasive devices	24 (36.9)	34 (52.3)	6 (9.2)	1 (1.5)	0
Continuous nocturnal glucose administration	8 (12.3)	25 (38.5)	23 (35.4)	3 (4.6)	6 (9.2)

Abbreviations: DK/NR, don’t know/no response.

**Table 2:** Sleep-related factors showing statistically significant differences in perceived relevance according to professional category.

	Highly Relevant n (%)	Relevant n (%)	Slightly Relevance n (%)	Not Relevant n (%)	DK/DR n (%)	*p*-Valor Mann-Whitney
NOCTURNAL ENVIRONMENTAL NOISE						
Nurses, n = 43	36 (83.7)	7 (16.3)	0	0	0	*p* = 0.001
Nursing assistants, n = 22	10 (45.5)	12 (54.5)	0	0	0	
PHYSICAL RESTRAINTS						
Nurses, n = 43	14 (32.6)	25 (58.1)	3 (7.9)	0	1 (2.3)	*p* = 0.011
Nursing assistants, n = 22	3 (13.6)	12 (54.5)	7 (31.8)	0	0	
DELIRIUM						
Nurses, n = 43	39 (90.7)	3 (7.0)	1 (2.3)	0	0	*p* < 0.001
Nursing assistants, n = 22	12 (54.5)	8 (36.4)	0	2 (9.1)	0	
CONTINUOUS NOCTURNAL GLUCOSE ADMINISTRATION						
Nurses, n = 43	2 (4.7)	14 (32.6)	19 (44.2)	3 (7.0)	5 (11.6)	*p* = 0.001
Nursing assistants, n = 22	6 (27.3)	11 (50.0)	4 (18.2)	0	1 (4.5)	

Abbreviations: DK/NR, don’t know/no response.

**Table 3:** Integration of Qualitative and Quantitative Findings on Nursing Staff Perceptions of Sleep in Critically Ill Pediatric Patients.

Qualitative Category and Subcategory	Main Qualitative Findings	Related Quantitative Findings
CATEGORY 1. FACTORS AFFECTING SLEEP. ENVIRONMENTAL FACTORS	Noise was identified as the primary factor disrupting sleep. Participants particularly highlighted monitor alarms, infusion pumps, conversations, and nighttime clinical activity. Artificial light and circadian rhythm disruption were also perceived as important contributors	Overall, 70.8% of participants rated noise as highly relevant. Artificial light exposure at night was considered highly relevant by 64.6% of participants, while 69.2% rated limited daytime exposure to natural light as highly relevant
CATEGORY 1. FACTORS AFFECTING SLEEP. CARE-RELATED FACTORS	Nursing interventions, nighttime procedures, and certain routine care practices were identified as common causes of sleep disruption. Participants emphasized the need to cluster nursing activities and increase flexibility in organizational routines	Overall, 72.3% of participants considered nighttime interventions a highly relevant factor contributing to sleep disturbances
CATEGORY 1. FACTORS AFFECTING SLEEP. CLINICAL FACTORS	Delirium, pain, and specific treatments were described as having a substantial impact on sleep quality in critically ill children	Pain was rated as highly relevant by 84.6% of participants, withdrawal syndrome by 87.7%, and delirium by 78.5%
CATEGORY 1. FACTORS AFFECTING SLEEP. PROTECTIVE FACTORS	Parental presence, individualized care, environmental control, and clustering of care activities were perceived as strategies that promote restful sleep	Limited family involvement was rated as relevant or highly relevant by 90.8% of participants.
CATEGORY 2. CONSEQUENCES OF SLEEP DISTURBANCES	Participants associated inadequate sleep with delayed recovery, an increased risk of complications, and the development of delirium	Delirium was among the factors perceived as most relevant (78.5%), reflecting a high level of awareness regarding its association with sleep disturbances
CATEGORY 3. PERCEPTION OF SLEEP IN THE PICU. OVERALL PERCEPTION	A widespread perception emerged that critically ill pediatric patients do not achieve adequate sleep during their PICU stay	Overall sleep quality was not directly assessed in the quantitative questionnaire; the quantitative component focused on the perceived relevance of specific sleep-disrupting factors
CATEGORY 3. PERCEPTION OF SLEEP IN YHE PICU. NEED FOR PROFESSIONAL AWARENESS AND IMPROVEMENT STRATEGIES	Participants identified the need to increase awareness of sleep promotion and to review specific clinical care routines	Significant differences between professional groups were observed in the perceived relevance of nocturnal environmental noise (*p* = 0.001), delirium (*p* < 0.001), physical restraints (*p* = 0.011), and continuous nocturnal glucose administration (*p* = 0.001)

Integrated interpretation: Qualitative and quantitative findings demonstrated a high degree of convergence. Both the interviews and the questionnaire identified noise, light, nighttime interventions, delirium, and other clinical factors as key contributors to sleep disturbances in critically ill pediatric patients. Likewise, both phases highlighted the need to increase professional awareness and to incorporate sleep promotion and protection as an explicit objectives of nursing care in the PICU.

## Data Availability

The data generated and analyzed during the current study are not publicly available due to ethical and confidentiality considerations but may be available from the corresponding author upon reasonable request and subject to approval by the research team.

## Abbreviations

The following abbreviations are used in this manuscript:

PICU	Pediatric Intensive Care Units
PICS-p	Pediatric Intensive Care Syndrome
TMSCAE	Nurses assistants (Spanish acronym)
IQR	Interquartile range
ICU	Intensive Care Unit
